# An American Medical Journal

**Published:** 1890-09-13

**Authors:** 


### THE PRACTITIONER'S MIRROR AND RETROSPECT.

### MEDICINE.

### AN AMERICAN MEDICAL JOURNAL.

The *American Journal of the Medical Sciences* is published monthly at Philadelphia, being edited by Dr. J. Minis Hays, and in a very recent number we notice the following articles: Dr. James Steward, professor of pharmacology and therapeutics, McGill University, Montreal, has a paper on tetany. The tetany of adult life is essentially an intermittent disease. The morbid manifestations are tonic spasms, frequently recurring for brief periods in one or other parts of the body. The disease is not associated with any recognised organic change, although persons of neurotic temperament seem to be specially liable. But the case related by Dr. Hays was also complicated with symptoms of myxœdema, and it appeared there was "a myxœdema engrafted on a tetany." We do not recollect that these maladies have been associated, but there appears no reason why they should not occur in the same individual.

We next have a paper on antipyrin in malarial fevers, by Dr. William T. Prout, colonial surgeon, Gold Coast, Africa. Dr. Prout states that in this drug we possess a remedy which is certain to cut short the duration of a paroxysm of malarial fever and which when used in combination with quinine shortens the whole attack. He also states that when given during the hot stage it affords almost immediate relief. Also that antipyrin is well retained by the stomach, an advantage, and by no means an unimportant one, especially in fevers where gastro-hepatic disturbances are an almost invariable concomitant. Large doses are most satisfactory. Thirty grains should be given at once, and repeated in an hour and a-half, and afterwards doses of fifteen grains may be given as required. During the interval quinine should be given. Dr. Prout thinks that the antipyrin reduces the temperature, thereby promoting the sweating stage which eliminates the *materies morbi*.

Dr. Theodore Diller follows next with some observations on the hereditary form of chorea, with report of a case. Dr. Diller refers to Koch's theory, which he regards as "very inviting." Koch says there exists a choreic virus or blood dyscrasia, which in some cases produces chorea and in others rheumatism, and in some both these affections. Now there appears little doubt that chorea must be classed among the so-called hereditary diseases. On the other hand there is equally little doubt that chorea may be induced in children who have no hereditary family history of the disease. Fright certainly has been the immediate exciting cause, and there is reason to believe the same may be said of worms; for chorea has ceased immediately on the expulsion of worms. Again want, neglect, ill-usage, weakness, and anæmia, are very common antecedents. An association between chorea and rheumatism has long been recognised. A large number of children suffering from chorea are found to have previously suffered from rheumatism. During chorea there is often a cardiac morbid murmur, and *post mortem* examination generally shows some morbid condition of the aortic, or mitral valves, or of both. We cannot, however, fully agree with Dr. Diller's endorsement of Koch's choreic virus or blood dyscrasia. There certainly appears to be hereditarily transmitted a proneness to certain maladies. In certain temperaments and constitutions, chorea occurs most frequently in families in which nervous diseases are traceable. But probably environment has quite as much to do with the excitation of chorea as heredity.

Next we have a paper by Dr. Sam. C. Busey, on the effusion of chyle and of chyle-like, milky, fatty, and oily fluids into the serous cavities. Cases are mentioned of effusion into the pleural cavities, or *chylo-thorax*; of effusion into the tunica vaginalis testis, or *galactocele*; and of chylus chyliform and oily ascites. Cases of the kind are mentioned at some length. But there is no special reference in Dr. Busey's paper to chyluria, which manifests itself by a milky appearance of the urine, and is associated with a microscopic nematoid entozoon in the blood; the *filaria sanguinis hominis*, first discovered by Dr. T. Lewis, of the Indian service. Neither does Dr. Busey mention the conditions known as *varix lymphaticus, lymph scrotum*, or *nævoid elephantiasis*, the characteristics of which are dilated lymphatic vessels followed by discharge of lymph, and often terminating in elephantiasis. It would have been interesting if Dr. Busey had told us whether or not the cases of *chylo-thorax*, of *galactocele*, and of *chylous ascites* were associated with the *filaria sanguinis hominis*.

The next paper is entitled "Urticaria pigmentosa," by Dr. Berry W. Stelwagon, of Philadelphia. A case is detailed of urticaria, with dark yellow pigmentation, occurring in a youthful blonde. The author considers that it is not the same malady as simple urticaria. He remarks that while many of the symptoms resemble true urticaria, in some respects they are so different that the identity of the two diseases can scarcely be admitted. And he regards this conclusion as strengthened by the fact that no intermediate cases (if the expression be allowable) are encountered. Now various forms of urticaria have been described, such as urticaria *evanida, perstans, conferta, subcutanea, tuberosa*, &c., but as a rule *urticaria pigmentosa* does not find a place in the classification. Dr. Stelwagon's case was characterised by vesicles and papules, rather than by the wheals of ordinary urticaria. We recollect, however, having seen yellow-tinted urticaria in connection with hepatic congestion, and it is possible the colour in Dr. Stelwagon's case may have originated from a jaundiced condition of the blood, which evidenced itself in the affected skin.

The remainder of the *American Journal of the Medical Sciences* is composed of reviews and notices of recent works, of extracts from British and Continental professional publications, and of (chiefly) American medical news. From the brief summary of the original articles as given above, it will be recognised that American professional literature is well to the front.

